# Immunological and Safety Considerations When Selecting the Dose Formulation of a Purified Inactivated Zika Virus Vaccine (PIZV)

**DOI:** 10.3390/microorganisms12071492

**Published:** 2024-07-21

**Authors:** Camilo J. Acosta, Francesco Nordio, Eloi Kpamegan, Kelley J. Moss, Pradeep Kumar, Kazuhiro Hirata

**Affiliations:** 1 Takeda Vaccines Inc., Cambridge, MA 02139, USA; francesco.nordio@takeda.com (F.N.); eloi.kpamegan@takeda.com (E.K.); kelley.moss@takeda.com (K.J.M.); 2 Takeda Pharmaceuticals International AG, 8152 Zürich, Switzerland; pradeep.kumar@takeda.com; 3 Takeda Pharmaceutical Company Limited, Osaka 541-0045, Japan; kazuhiro.hirata@takeda.com

**Keywords:** PIZV, TAK-426, Zika virus, purified inactivated Zika vaccine, dose selection, safety, neutralizing antibody response, cross-reactive immunity, vaccine

## Abstract

We previously reported the first-in-human assessment of three doses (2, 5, and 10 µg) of purified inactivated Zika virus vaccine (PIZV or TAK-426) in the Phase 1 ZIK-101 study (NCT03343626). Here, we report dose selection based on extended safety and immunogenicity data (6 months post-vaccination) and discuss considerations (e.g., immunological, historic, flavivirus immunological cross-reactions) for selecting a Zika virus (ZIKV) vaccine dose formulation. TAK-426 dose selection was conducted at the first interim analysis, and was based on cumulative safety data from both flavivirus-naïve (up to ≥28 days post-dose PD2) and flavivirus-primed participants (up to ≥28 days PD1), and on immunogenicity data from flavivirus-naïve participants only (at 28 days PD1 and 28 days PD2). The safety profile from TAK-426 recipients was compared to placebo recipients. Immunogenicity was assessed by geometric mean titer ratios of neutralizing anti-ZIKV antibodies and differences in seroconversion rates. There was no significant difference in safety between the three TAK-426 doses. The 10 μg dose provided the earliest and strongest immune response (with close to 100% seroconversion and higher antibody titers PD1 in flavivirus-naïve participants), and was well tolerated with acceptable safety profiles in both flavivirus-naïve and flavivirus-primed participants; this dose was selected for further development.

## 1. Introduction

Zika virus (ZIKV) is a single-stranded positive-sense RNA virus that is primarily a mosquito-borne flavivirus (FV). It was first isolated in 1947 from a rhesus monkey in the Zika Forest of Uganda [1,2]. The primary mode of ZIKV transmission is through the bite of infected female mosquitoes, prominently the *Aedes aegypti* and *Aedes albopictus* species that have spread globally [3]. Other recognized modes of transmission are mother-to-fetus during pregnancy, mother-to-child during the perinatal period, and sexual contact; few cases have been reported from blood component transfusion [3,4,5,6,7,8,9]. Exposure to ZIKV through laboratory work, solid organ transplants, and body fluids (e.g., tears, urine, breastmilk) are other potential mechanisms of disease transmission; however, in each of these cases, there is limited evidence [10,11,12,13,14,15]. The incubation period between exposure and the development of symptoms associated with ZIKV infection is approximately 3 to 11 days [16,17].

Available data strongly endorse links between ZIKV infection and congenital anomalies (congenital ZIKV syndrome) [18,19], as well as between ZIKV infection and Guillain–Barré syndrome (GBS) [19,20,21,22,23,24]. Congenital ZIKV syndrome is characterized by severe microcephaly, subcortical calcifications, macular scarring, focal pigmentary retinal mottling, congenital contractures, and early hypertonia [25]. GBS is characterized by a short time to onset following infection and a rapid evolution of the disease [26]. The pathogenesis of GBS in association with ZIKV infection is not understood, and the long-term clinical course is unknown.

Because of the low but sustained ZIKV transmission in endemic countries, as well as the risk of reemergence in areas with prior transmission, the development of a Zika vaccine is considered crucial to protect susceptible populations, including women of child-bearing potential living in and travelers visiting Zika-endemic areas [27,28,29,30]. Several platform technologies for ZIKV vaccine development have been employed: deoxyribose nucleic acid vaccines, messenger RNA vaccines, purified inactivated virus vaccines, and adenovirus-based vaccines. Takeda has developed an aluminum hydroxide-adjuvanted purified inactivated Zika virus vaccine (PIZV) candidate, also known as TAK-426, for the target indication of prevention of disease caused by ZIKV. In preclinical testing, TAK-426 was safe and well tolerated in New Zealand white rabbits (data on file), elicited robust immune responses, and protected mice and Indian rhesus macaques in a ZIKV challenge model [31,32].

An inactivated ZIKV vaccine cannot revert to a virulent form that is capable of causing and transmitting disease. Additionally, inactivated vaccines are not contraindicated during pregnancy. An inactivated ZIKV vaccine would be well suited and safer for the control of the current low and sustained transmission of ZIKV in endemic areas.

Immunogenicity data in two mice models (CD-1 and AG-129) and efficacy data in AG-129 mice demonstrated that two doses of TAK-426 formulated with an aluminum hydroxide adjuvant elicited robust immune responses [31]. Two doses of TAK-426, administered 28 days apart, have so far been shown to be well tolerated in over 100 participants with an acceptable safety profile in both FV-naïve and FV-primed healthy adults aged 18–49 years. TAK-426 induced a dose-dependent humoral immune response in both FV-naïve and FV-primed participants [33]. TAK-426 also induced neutralizing antibody (nAb) magnitudes and kinetics that appear to be comparable to those elicited by ZIKV natural infection [34]. Previously, we described the selection of the TAK-426 vaccine dose formulation during Phase 1 studies based on characterization of the immunogenicity dose–response in FV-naïve adults only and characterization of the safety profile in FV-naïve and FV-primed adults who received at least one vaccine dose [33]. We now present and discuss the dose selection with the extended safety follow-up and the TAK-426-induced immunogenicity data 6 months after vaccination with low (2 μg) and medium (5 μg) doses, as well as with a high (10 μg) dose that was selected for further clinical development. We also discuss how historic considerations of other inactivated virus vaccines and flavivirus immunological cross-reactions ought to be considered when selecting a ZIKV vaccine regimen and dose.

## 2. Materials and Methods

This Phase 1, randomized, observer-blind, placebo-controlled ZIK-101 study was the first trial of TAK-426 in humans. The dose-selection study assessing the safety and immunogenicity of TAK-426 enrolled FV-naïve and FV-primed healthy adults aged 18 to 49 years from the United States and Puerto Rico [33,34]. Due to known cross-reactivity between FVs, FV-naïve and FV-primed participants were sequentially enrolled. The ZIK-101 protocol was approved by the local ethics committee or institutional review board of each study center, registered on ClinicalTrials.gov (NCT03343626), and implemented in accordance with International Council for Harmonisation, Good Clinical Practice guidelines, the Declaration of Helsinki, and applicable local regulatory requirements.

A total of 271 participants were enrolled and randomized (1:1:1:1) into four groups: placebo (saline solution), and 2, 5, or 10 µg of TAK-426. Each dosing group comprised more than 60 participants, with at least 30 participants in the FV-naïve cohort and 30 in the FV-primed cohort. A total of 125 participants were enrolled in the FV-naïve cohort and 146 participants in the FV-primed cohort. All participants were followed for a minimum of 6 months post-dose 2 (PD2) for the assessment of safety and persistence of the vaccine-induced immune response. Although the placebo group and the selected dose group (10 µg) were followed for up to 24 months PD2 for the assessment of the persistence of immunity and long-term safety, only the safety and immunogenicity data 6 months after vaccination were used to discuss dose selection. An independent Data Monitoring Committee had the safety oversight of this study.

Flavivirus antibody status at the time of screening of participants was performed utilizing a Multiplex Luminex^®^ IgG enzyme-linked immunosorbent assay (Luminex Corp, Austin, TX, USA). The assay included multiple relevant flavivirus antigens, which were conjugated to Luminex beads (Table 1).

The vaccination regimen consisted of two doses of TAK-426 administered intramuscularly 28 days apart. The primary objectives of this Phase 1 study were as follows: (1) to describe the safety of two doses of TAK-426 with three different antigen dose levels (2, 5, or 10 μg) in FV-naïve and FV-primed healthy adults through 28 days PD2; and (2) to select a single dose level from three different antigen concentrations (2, 5, or 10 µg) of TAK-426 for further clinical development.

The primary rationale for selection of the three vaccine dose levels was as follows: (1) the low-dose level (2 µg) was selected for purposes of potentially identifying an immunological threshold, and was based on the limit of the analytical methods for the drug product available at the time; (2) the mid-dose level (5 µg) was selected to provide a mid-point on the dose–response curve. In addition, this dose level was based on a Phase 1 study of the Walter Reed Army Institute of Research Zika vaccine candidate (ZPIV) [35], which was originally based on initial studies in mice and non-human primates; and (3) the high-dose level (10 µg) was selected to follow an approximate 2-fold range and to further define the potential dose–response curve in both FV-naïve and FV-primed participants.

### 2.1. Safety Assessments

Safety assessments for the ZIK-101 study have been previously described [33,34]. Following earlier assessments of solicited local and systemic AEs (7 days after each dose) and unsolicited AEs (28 days after each dose) [33], serious AEs (SAEs) and new medical conditions (including neurological and neuroinflammatory disorders) were reported through the 2-year follow-up [34].

### 2.2. Acceptability Assessment of the Safety Profile

The acceptability of the safety profile was based on the comparison to the placebo (saline) group. In addition, marketed inactivated aluminum hydroxide-adjuvanted vaccines (the most relevant being FV vaccines) were also used for comparison, particularly for the injection site reactions (local reactogenicity) [36,37,38,39]. The acceptability of the safety profile (PD1 and PD2) was determined not only based on the frequency of the relevant safety parameters (solicited events, unsolicited events, and safety laboratory parameters), but also on other parameters that characterized the AE(s), such as nature, latency, duration, outcome, potential impact on public health or the healthcare system (e.g., ER visits, hospitalizations), safety laboratory parameters, and change from baseline. Any increase in reactogenicity PD2 also impacted the assessment of the safety profile of the vaccine. In case any related SAE and/or any SAE leading to withdrawal of the investigational medicinal product occurred, the acceptability of the safety profile of a vaccine dose relied on the detailed analyses of such (S)AEs. If it appeared that such events were segregated to one vaccine dose level with unclear causality to the study vaccine, then there may not be an acceptable safety profile at that dose level.

### 2.3. Immunogenicity Assessments

To evaluate post-vaccination immune responses, serum samples for all participants were obtained at baseline (day 1) and at 1, 2, and 8 months. Anti-ZIKV nAb levels were measured using a qualified plaque reduction neutralization test (PRNT) at Q2 Solutions (San Juan Capistrano, CA, USA) The Zika PRNT test had a lower limit of detection of 1:10 dilution and a lower limit of quantitation of 26 (reciprocal dilution) [33,34]. ZIK-101 serum ZIKV NAb levels were also measured using a fit-for-purpose ZIKV reporter virus particle (RVP) assay, developed and performed in the Takeda Laboratory in Cambridge, Massachusetts, with a lower limit of quantitation of 105, which was used as the threshold to define seropositivity or seronegativity.

The assessment of TAK-426 immunogenicity data from FV-naïve and FV-primed participants occurred at 28 days PD1, 28 days PD2, and 6 months PD2. 

### 2.4. Dose Selection (Phase 1 Interim Analysis)

As previously described [33], dose selection was based on the magnitude of the immune response in FV-naïve participants, as measured by the ratios of GMTs of anti-ZIKV nAbs and differences in the seroconversion rate between the dosing groups, as well as safety outcome measures of FV-naïve and FV-primed participants. The interim analyses were performed by a separate set of unblinded statisticians and programmers at a Clinical Research Organization, who had access to individual treatment assignments. All personnel involved in the conduct of the trial, including those at Takeda, the Clinical Research Organization, and the trial sites, remained blinded to the individual participant treatment assignment. The study team had access to the group level unblinded results only. The preliminary TAK-426 efficacy (extrapolated from preclinical anti-ZIKV nAbs associated with protection against a ZIKV challenge in non-human primates [rhesus macaques]) was also taken into consideration as supplemental information in dose selection [40].

The final dose-selection decision was made following the pre-specified statistical analysis plan and its consistency with one of the four different qualitative scenarios described in Table 2.

### 2.5. Statistical Analysis

The sample size was not determined by formal statistical power calculations. However, stochastic simulations (with 1 million simulation runs) suggested that 60 participants per group were adequate to select one of the three tested doses by the ratios of geometric mean titers (GMTs) between the dosing groups [33]. With 60 participants in each group, the probability of observing a common adverse event (AE) (10% true rate) was over 80%. Safety assessments were performed on all randomized participants who received ≥1 dose of vaccine or placebo (safety set). Safety endpoints are summarized descriptively with frequency and percentage for categorical data. The number and percentage of participants with at least 1 solicited local and systemic AE are reported. For participants with more than 1 episode of the same event, the maximum severity is used for tabulations. Unsolicited AEs, up to 28 days after each injection, are coded using MedDRA and summarized by SOC and PT by trial arm. Safety summaries are provided overall and by severity (solicited AEs). Immunogenicity assessments were based on the per-protocol set, comprising all participants with no major protocol violations who received ≥1 dose of the investigational vaccine or placebo and provided valid baseline serology and ≥1 postvaccination time point. Anti-ZIKV antibody levels were compared between the dosing groups using GMTs ratios and seroconversion rate (SCR) differences. The point estimates of GMT ratios ≥2 were considered meaningful to differentiate the immunogenicity in terms of GMTs between different dose levels. Seroconversion was defined as seronegative participants at baseline who became seropositive after vaccination or seropositive participants at baseline with ≥4-fold increases in GMTs. For the calculation of GMTs, seronegative samples were assigned a titer of 5 (half of the limit of detection), and the 95% confidence intervals (CIs) were calculated using the exact Clopper–Pearson method for each dosing group and time point. ANOVA analysis of immunogenicity as measured by the pairwise ratios of GMT of anti-ZIKV nAbs were also performed. The distribution of neutralization titers of each dose level was also evaluated using reverse cumulative distribution curves (RCDCs), and comparisons were made based on individuals reaching no less than 70% of high anti-ZIKV nAb titers. We used SAS, version 9.2, for statistical analyses.

## 3. Results

FV-naïve cohort (n = 125) PD1 and PD2 safety data as well as FV-primed cohort (n = 146) PD1 data only were used to select the TAK-426 dose for further vaccine development. This early selection based on PD1 data allowed for the initiation of manufacturing Phase 2 clinical trial materials. Demographic characteristics and disposition of participants of both cohorts were reported previously [33]. Notably, no TAK-426-related SAEs were reported in any FV cohort.

### 3.1. FV-Naïve Cohort

**Figure 1:**
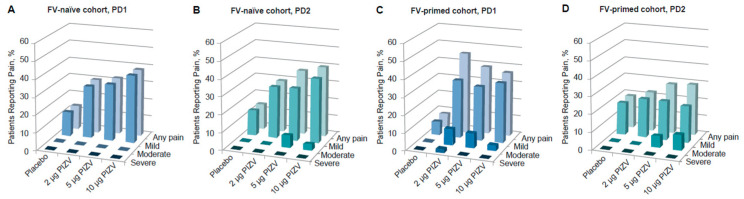
Incidence of solicited local pain in the FV-naïve cohort PD1 (**A**) and PD2 (**B**), and in the FV-primed cohort PD1 (**C**) and PD2 (**D**). FV: flavivirus, PIZV: purified inactivated Zika virus vaccine, PD: post-dose.

Solicited local reactions and systemic AEs occurring during the 7 days after each dose were previously reported [33]. Overall, the reporting rates of the total solicited AEs (local and systemic after both doses) in the TAK-426 groups were comparable to those reported in the placebo group. While solicited local AE rates were higher among those vaccinated with TAK-426 as compared to the placebo group, systemic AE rates were higher among placebo recipients (as compared to TAK-426 vaccinees). Pain at the injection site was the most frequent solicited AE, with no significant difference between doses, but slightly higher pain incidence rates were reported with increasing TAK-426 dose levels after the first and second doses. TAK-426 10 µg induced pain reactions were not severe (Grade 3, as defined by the FDA Toxicity Grading Scale) (Figure 1). The only Grade 3 (severe) solicited AE, fever, was reported in the placebo group PD2 (Table 3). Unsolicited AEs were higher among placebo recipients, with the lowest rate reported by those receiving the 10 µg TAK-426 dose (19% vs. 42% in the placebo group).

### 3.2. FV-Primed Cohort

Solicited local reactions and systemic AEs occurring during the 7 days PD1 of TAK-426 were reported previously [33]. In summary, the reporting rates of the total solicited AEs (local and systemic after both doses) in the TAK-426 groups were higher than those reported in the placebo group. PD1, the reporting rates of solicited local AEs were higher in the TAK-426 groups than in the placebo group (45%, 42%, and 37% in the 2 µg, 5 µg, and 10 µg TAK-426 groups, respectively, vs. 14% in the placebo group). A similar reporting pattern was observed PD2, albeit with lower rates, as compared to PD1 in all TAK-426 groups (22%, 31%, and 32% in the 2 µg, 5 µg, and 10 µg TAK-426 groups, respectively, vs. 18% in the placebo group). Systemic AE rates were comparable or lower than placebo PD1 (24%, 36%, and 37% in the 2 µg, 5 µg, and 10 µg TAK-426 groups, respectively, vs. 33% in the placebo group) but higher than or comparable to placebo PD2 (19%, 31%, and 21% in the 2 µg, 5 µg, and 10 µg TAK-426 groups, respectively, vs. 21% in the placebo group). Like the FV-naïve cohort, previously FV-exposed individuals reported fewer unsolicited AEs in the TAK-426 groups (27%, 30%, and 22%, respectively) as compared to the placebo (31%), with the lowest rates reported by those receiving the 10 µg TAK-426 dose (22%).

Pain at the injection site was the most frequent solicited AE reported in both FV-naïve and FV-primed cohorts. Most of these events were reports of mild to moderate pain at the injection site, with onset on day 1 and with a mean duration of less than 2 days. There was one pain case only, in a FV-primed participant, described as severe following the first dose of TAK-426 2 µg (see Table 3). As shown in Figure 1, among FV-naïve participants, the reported rates of pain after any dose were slightly higher in those receiving the TAK-426 10 µg dose as compared to 2 µg and 5 µg; the opposite was reported among FV-primed individuals following the first dose, whereby the lowest rate was reported in those receiving the highest dose.

A total of 22 Grade 3 (severe) solicited and unsolicited AEs were reported, with most (20 out of 22) reported by FV-primed participants. Most Grade 3 AEs were reported among those receiving TAK-426 5 µg (n = 10), followed by the 2 µg (n = 6), 10 µg (n = 4), and placebo (n = 2) groups. Details on the Grade 3 AEs by dose are provided in Table 3. No deaths and no Grade 4 AEs were reported in the FV-naïve or the FV-primed cohorts. There were no safety laboratory changes of concern at 7 days post-vaccination in any TAK-426 group in either cohort. Most blood chemistry and hematology values remained within normal limits or were not above Grade 1. No participants in the FV-naïve cohort and two participants in the FV-primed cohort had Grade 4 prothrombin time (>1.25 upper limit of normal). Of the two participants with Grade 4 prothrombin time (PT), one participant received TAK-426 (5 µg) and the other participant received placebo. PTs of both participants were within normal ranges on day 57.

In summary, among participants previously exposed to FVs, there were some apparent differences in the safety profile between doses, favoring TAK-426 10 µg; the lower the TAK-426 dose, the higher the rates of solicited AEs PD2 and unsolicited AEs after any dose. Similarly, the lower the dose, the higher the number of Grade 3 (severe) solicited AEs, which occurred more often in FV-primed than in FV-naïve participants.

### 3.3. TAK-426-Induced Immune Responses

TAK-426 was immunogenic in both FV-naïve and FV-primed adults aged 18 to 49 years. TAK-426 2 µg and 5 µg recipients were followed up to 6 months PD2, and TAK-426 10 µg recipients up to 24 months PD2, as previously reported [34]. Briefly, TAK-426 induced a dose-dependent humoral immune response in both FV-naïve and FV-primed participants. TAK-426-induced ZIKV nAbs persisted for up to at least 24 months among FV-naïve individuals and up to at least 12 months in FV-primed individuals. Anti-ZIKV nAb titers declined after 6 months in both cohorts. While FV-naïve ZIKV nAbs reached a steady state from 12 to 24 months PD2, FV-primed ZIKV nAbs continued dropping to levels similar to those in the placebo group (95% CIs overlapped). FV-naïve seropositivity rates (SPRs) and SCRs were high (>94%) and remained elevated up to 24 months PD2. FV-primed SPRs remained at 100% up to 12 months and declined to 76% by 24 months PD2.

### 3.4. FV-Naïve TAK-426-Induced nAbs Comparison (2 µg, 5 µg, and 10 µg)

**Figure 2:**
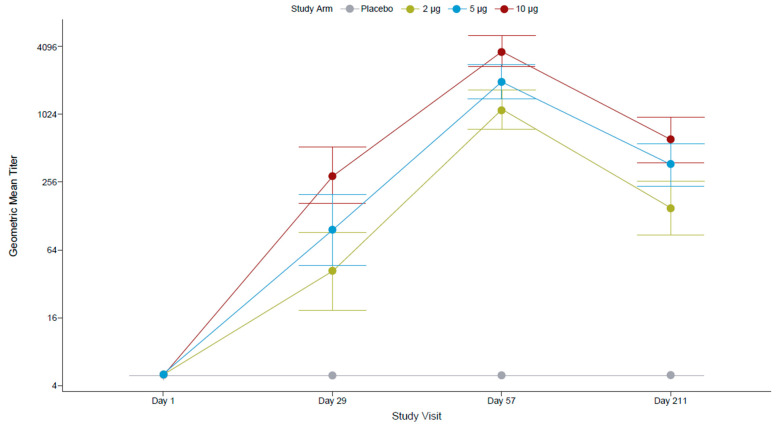
TAK-426-induced nAbs in the FV-naïve cohort by TAK-426 dose (2 µg, 5 µg, and 10 µg). GMTs (PRNT) and 95% CIs are reported at baseline, PD1 (day 29), 28 days PD2 (day 57), and 6 months PD2 (day 211) for the per-protocol set. FV: flavivirus, GMT: geometric mean titer, PD: post-dose, PRNT: plaque reduction neutralization test.

All FV-naïve participants at screening were seronegative for antibodies against FVs (IgG ELISA assessing 12 FV antigens) and for anti-ZIKV nAbs (by PRNT). After the first dose, most participants (96.4%) who had received the 10 µg TAK-426 dose had seroconverted (titer ≥ 10), compared to 82.1% in the 5 µg TAK-426 group and 72.0% in the 2 µg TAK-426 group. The second dose increased the GMTs by over 10 times in all TAK-426 groups (Figure 2). The placebo group remained seronegative throughout the study.

Pairwise comparison of PRNT GMTs (Table 4) showed no statistically significant differences between TAK-426 groups PD1, whereas at 1 month PD2, the GMT of the 10 µg TAK-426 group was significantly higher than the GMT of the 2 µg TAK-426 group (3.27 times higher; *p* < 0.001) and the GMT of the 5 µg TAK-426 group (1.85 times higher; *p* = 0.012); the GMT of the 5 µg TAK-426 group was significantly higher than the GMT of the 2 µg TAK-426 group (1.76 times higher; *p* = 0.027). The 95% CIs around the GMTs did not overlap between the 2 µg and 10 µg TAK-426 groups at either time point. Comparable pairwise results were observed with the reporter virus particle (RVP) assay.

Anti-ZIKV nAb titers declined after 6 months (a reduction of 87%, 82%, and 84% in the 2 µg, 5 µg, and 10 µg TAK-426 groups, respectively), though SCRs remained at 100% in all groups. Similar to GMT ratios at 1 month PD2, at 6 months, the GMT of the 10 µg TAK-426 group was significantly higher than the GMT of the 2 µg TAK-426 group (GMT ratio: 3.9; 95% CI: 2.2–7.1; *p* = 0.0004) and 2-fold higher than the GMT of the 5 µg TAK-426 group (GMT ratio: 1.9; 95% CI: 1.1–3.4; *p* = 0.1160).

**Figure 3:**
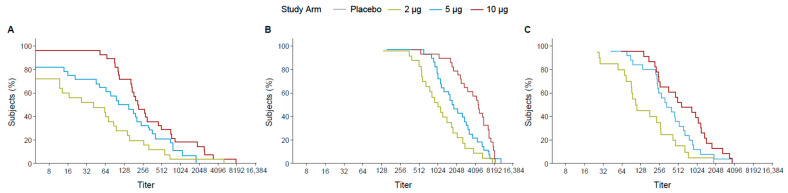
RCDCs of FV-naïve ZIKV nAbs 28 days PD1 (day 29) (**A**), 28 days PD2 (day 57) (**B**), and 6 months PD2 (day 211) (**C**). FV: flavivirus, PD: post-dose, PRNT: plaque reduction neutralization test, RCDC: reverse cumulative distribution curve.

Following the first dose, the 10 µg TAK-426 group had 70% of participants with titers ≥ 128 while the 5 µg TAK-426 and the 2 µg TAK-426 groups had 70% of the participants with titers ≥ 64 and ≥16, respectively. The difference was greater at 2 months PD2, where 70% of the participants had titers ≥ 2048, ≥1024, and ≥512 in the 10, 5, and 2 µg groups, respectively. After 6 months following the second dose, the 10 µg and the 5 µg TAK-426 groups had similar antibody levels with 70% of the participants having titers ≥ 256, while titers in the 2 µg group dropped to ≥64 (Figure 3).

### 3.5. FV-Primed TAK-426-Induced nAbs Comparison (2 µg, 5 µg, and 10 µg)

All FV-primed participants at screening were seropositive for antibodies against FVs (IgG ELISA assessing 12 FV antigens), and most (81.3%) had anti-ZIKV nAbs (PRNT). After the first dose, 100% of participants who received the 5 µg and the 10 µg TAK-426 dose, and 97% who received the 2 µg dose had nAbs against ZIKV. The highest seroconversion rates of PD1 and PD2 were reported for the 10 µg TAK-426 group (70% and 77%, respectively), as compared to the 5 µg (50% for PD1 and 55% for PD2) and 2 µg (39% for PD1 and 47% for PD2) groups. A limited number of participants in the placebo group seroconverted during the follow-up (3% PD1 and 6% PD2).

**Figure 4:**
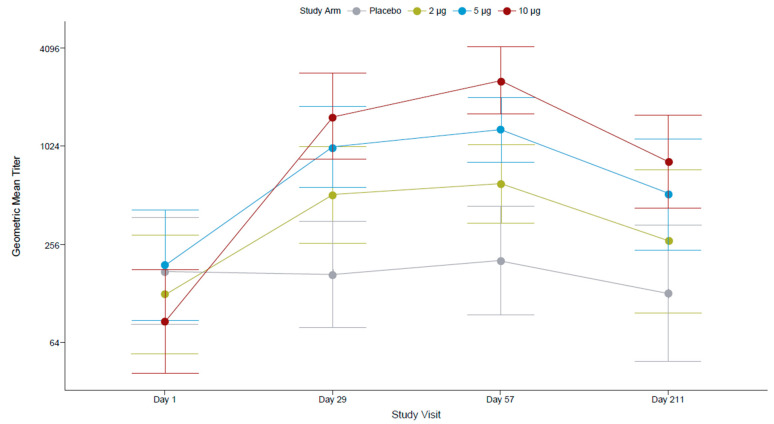
TAK-426-induced nAbs in the FV-primed cohort by TAK-426 dose (2 µg, 5 µg, and 10 µg). GMTs (PRNT) and 95% CIs are reported at baseline, PD1 (day 29), 28 days PD2 (day 57), and 6 months PD2 (day 211) for the per-protocol set. FV: flavivirus, GMT: geometric mean titer, PD: post-dose.

TAK-426 10 µg dose 1 (day 29) elicited the highest (21-fold) increase from baseline ZIKV nAb titers as compared to the 2 µg (4-fold) and the 5 µg (5-fold) groups. The magnitude of the increase in GMTs PD2 was <2-fold (1.6 for 10 µg, 1.3 for 5 µg, 1.2 for 2 µg) as compared to the increase in GMTs PD1 (≥4-fold). While the 95% CIs around the GMTs overlapped between all TAK-426 dosing groups at 28 days PD1, they did not overlap between the 2 µg and 10 µg TAK-426 groups at 28 days PD2 (Figure 4).

In the 10 µg TAK-426 group, GMTs decreased at 6 months PD2 (866.26, 95% CI: 445.59–1684.08) as compared to 28 days PD2 (2590.54, 95% CI: 1649.18–4069.22). However, they remained 11-fold higher than at baseline (73.46, 95% CI: 35.59–151.62). The fold increase from baseline to 6 months PD2 was 2 for the 2 µg group and 3 for 5 µg group; no fold increase was observed in the placebo group. The decline in anti-ZIKV nAb titers at 6 months PD2 was 57%, 61%, and 67% in the 2 µg, 5 µg, and 10 µg TAK-426 groups, respectively.

The GMTs overlapped between the 5 µg and 10 µg TAK-426 groups, suggesting they were comparable at either time point up to 6 months PD2 (GMT ratios ≤ 2). The GMT ratios between the 2 µg and 10 µg TAK-426 groups were 3.1 (95% CI: 1.3–7.1; *p* = 0.01) at 28 days PD1, 3.7 (95% CI: 2.0–6.8; *p* < 0.0001) at 28 days PD2, and 2.2 (95% CI: 1.1, 4.8; *p* < 0.05) at 6 months PD2.

**Figure 5:**
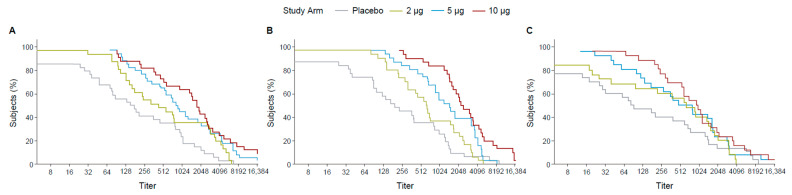
RCDCs of FV-primed ZIKV nAbs at 28 days PD1 (day 29) (**A**), 28 days PD2 (day 57) (**B**), and 6 months PD2 (day 211) (**C**). FV: flavivirus, PD: post-dose, PRNT: plaque reduction neutralization test, RCDC: reverse cumulative distribution curve.

The RCDCs indicated that across all dose groups at three time points (1 month PD1, 1 month PD2, and 6 months PD2), 70% of the participants achieved titers ≥ 256. The curves overlapped for titers ≥ 512 (Figure 5).

## 4. Discussion

Phase 1 safety and immunogenicity data supported the selection of two doses of TAK-426 10 µg administered 28 days apart for further clinical development. The dose selection was based mainly on data from FV-naïve participants and was reached following execution of a pre-specified interim analysis plan that generated data meeting pre-defined safety and immunogenicity criteria consistent with decision-making scenario 1 described in Table 2. The key decision driver was TAK-426-induced humoral immune response differentiation, leading to the selection of the highest antigen content dose (10 µg). The Phase 1 dose-selection decision was supported by safety and immunogenicity data, as well as by predicted efficacy data [40], up to 6 months PD2 in the FV-naïve cohort.

One limitation of our early dose selection is that most of the FV-primed safety and immunogenicity data were not included (as per protocol). These FV-primed data may fit best with the second decision-making scenario (Table 2) in which the major decision driver would be safety, because, as compared to FV-naïve participants, FV-primed adults reported more severe solicited systemic AEs than the placebo group. Among FV-primed participants, there were more Grade 3 (severe) solicited systemic events PD2 (10 events) than after the first dose (four events). Most FV-primed adults were residents in Puerto Rico and therefore most likely had been exposed to dengue virus (DENV) prior to, during, and/or after trial enrollment. DENV cross-reactive immune responses might have contributed to such apparent AE increases. FV-primed data seemed to indicate that higher doses of the TAK-426 antigen led to less severe AEs, supporting the early selection of the 10 µg antigen content.

The FV-primed data also suggested that a single TAK-426 5 µg or 10 µg dose would suffice since they both induced comparable ZIKV nAb titers 28 days PD1, in which case a second dose would not be required. Indeed, a single 5 µg dose of another Zika-inactivated vaccine (ZPIV), albeit less immunogenic than TAK-426, was sufficient to elicit cross-reactive neutralizing antibodies against ZIKV and DENV in a participant with prior DENV exposure [41]. TAK-426 5 µg could induce fewer severe AE cases as compared to a single dose of 2 µg (Table 3 and scenario 4 in Table 2). A single TAK-426 5 µg dose, administered to FV-primed individuals (i.e., participants with prior DENV natural infection), would have a similar safety profile as a single TAK-426 10 µg dose with some manufacturing advantages and less cost. However, the benefit of a booster dose to ensure long-term protection, if not achieved through natural exposure, should also be explored.

Injection-site pain was the most frequently reported solicited AE during the ZIK-101 study. Participants reported pain more frequently than those who received other licensed FV vaccines such as YF-VAX^®^ (<5% in uncontrolled clinical trials) [38], TICOVAC (13.2% in adults 16-65 years old) [39], and IXIARO (>25% in adults > 18 years-old) [37]. Pain was reported less frequently in the ZIK-101 study compared to another trial of a Zika-inactivated candidate vaccine (ZPIV) [42].

Two precedents challenge our ZIKV dose-selection approach assumption that the human dose–response to an inactivated ZIKV vaccine is saturating. Firstly, past studies of virus-inactivated vaccines have shown that low-antigen content candidate vaccines developed for mycoplasma pneumoniae [43] and for other infectious diseases (e.g., Japanese encephalitis, Rocky Mountain spotted fever, typhus, and lymphocytic choriomeningitis) [44] led to vaccine-associated enhancement of the target disease (homotypic vaccine-associated enhanced disease [VAED]) in humans and animal models. This increase in homotypic VAED rates was also reported for high-antigen content candidate vaccines developed to prevent trachoma [45], respiratory syncytial virus [46,47], and measles virus [48]. So far, very limited published data support the occurrence of homotypic ZIKV immune enhancement [49,50]. In contrast, heterotypic FV-immune enhancement has been reported in humans for DENV [51,52,53], ZIKV [54], and West Nile virus [55]. Heterotypic VAED has also been observed with an inactivated dengue vaccine candidate followed by a live-attenuated booster [56] and a licensed attenuated dengue vaccine [57]. Secondly, past dengue studies have demonstrated that a range of FV antibody concentration (dose-dependent in vaccinees) is a key factor predicting dengue disease enhancement [53]. Dengue disease enhancement has been studied in humans [58], non-human primates [59], in vitro experiments [60,61], and molecular simulations [62]. It remains unknown if TAK-426 could induce homotypic or heterotopic VAED. These two precedents indicate that a peaked dose–response curve model may best fit the TAK-426-induced immune responses, as seen in HIV [63], influenza [64], and malaria [65] vaccines. If so, a ZIKV vaccine candidate selection should not be based purely on high antibody responses but also on the likelihood of causing VAED. Although the ZIK-101 study was not designed or powered to determine safety differences across dosages, the higher total number of severe solicited AEs observed in the 2 µg dose group (3 AEs) compared to the 5 µg and 10 µg dose groups (1 AE in each dose group) of FV-primed participants PD1 could be an early indicator of a potential low-dose VAED.

In the case of Zika, two recent FV cross-reaction events are also to be factored in when developing and selecting the regimen and the dose for any ZIKV vaccine: the observed increase in the risk of severe dengue disease after natural ZIKV infection [54], and the lower Zika risk in dengue-seropositive recipients of a dengue vaccine [66]. When planning future TAK-426 development steps, FV immunological cross-reactions (in particular those that enhance or protect against DENV or ZIKV) are to be leveraged.

Zika antigen content (dose) is therefore a critical PIZV modifiable vaccine attribute likely to predict a better safety profile (i.e., less likely to induce homotypic and/or heterotopic VAED). The tendency to select a vaccine candidate based purely on high antibody responses may have been due to the association in early studies of low-antigen content with enhanced disease; for instance, when it was noted in a publication for the polio vaccine that “with a low antigen killed vaccine you stand the danger of actually doing more harm than good” [44]. Caution should be exercised now that fractional doses of licensed vaccines are recommended and being used [67]. The other critical safety-related modifiable vaccine attribute is the actual inactivation manufacturing process. Such inactivation processes should preserve the epitopes necessary to induce an optimal (e.g., neither the lowest nor the highest) immune response. With the current worldwide use of inactivated vaccines to protect millions of people against several infectious diseases, and with the accumulated TAK-426 data and learnings from the ZIK-101 study, we are cautiously optimistic and more confident on the next steps toward the development of an inactivated vaccine against ZIKV.

## Figures and Tables

**Table 1:** Antigens included in the initial Multiplex Luminex^®^ assay for detection of flaviviral antibodies (IgG) in serum or plasma samples for the Phase 1 study.

	ZIK-101 Study Antigen Description
1	Zika Virus NS1 (Uganda strain)
2	Zika Virus NS1 (Suriname strain)
3	Zika Virus VLP (E, prM/M)
4	Dengue Virus Serotype 1 VLP
5	Dengue Virus Serotype 2 NS1
6	Dengue Virus Serotype 3 NS1
7	Dengue Virus Serotype 4 VLP
8	Usutu Virus NS1
9	Yellow Fever Virus NS1
10	West Nile Virus NS1
11	West Nile Virus E (Domain III)
12	St. Louis Encephalitis Virus NS1

Abbreviations: E: envelope, IgG: immunoglobulin G, NS1: non-structural protein 1, prM/M: pre-membrane/membrane, VLP, virus-like particle.

**Table 2:** Decision-making scenarios for dose selection.

	Assessment Outcome	Dose Selection Based on
Scenario 1	Acceptable safety profile across all dose levels with difference in immunogenicity between dose levels	Immune response differentiation
Scenario 2	Comparable immunogenicity between dose levels with difference in safety profile	Safety profile differentiation
Scenario 3	Difference in both immunogenicity and safety profile between dose levels	Safety and immunogenicity profiles differentiation
Scenario 4	Acceptable safety profile across all dose levels with no difference in immunogenicity	Minimal dose with adequate immune response ^a^

^a^ Determination of the ‘adequate’ immune response will be based on the preliminary CoP and TAK-426 efficacy determined in non-human primates. Abbreviation: CoP: correlate of protection.

**Table 3:** Total number of Grade 3 (severe) solicited and unsolicited AEs by cohort (FV-naïve or FV-primed) and TAK-426 dose.

		PD1		PD2		Total	
		FV-Naïve n = 31	FV-Primed n = 36	FV-Naïve n = 31	FV-Primed n = 37	FV-Naïve n = 125	FV-Primed n = 146
Solicited AEs	Local	0	1 Pain (2 μg)	0	0	0	1
	Systemic	0	2 Headache (2 μg and 5 μg) 2 Myalgia (2 μg and 10 μg)	1 Fever (Placebo group)	2 Myalgia (5 μg and 10 μg) 1 Malaise (5 μg) 5 Fever (5 μg) 1 Headache (2 μg) 1 Arthralgia (5 μg)	1	14
Unsolicited AEs	Any	0	1 Transient ischemic attack (2 μg group)	1 Postpartum hemorrhage (2 μg group)	1 Blood fibrinogen increase (5 μg) 1 Colitis (10 μg) 1 Major depression (Placebo) 1 Acute cholecystitis (10 μg)	1	5
	Related	0	0	0	0	0	0

Abbreviations: AE: adverse event, FV: flavivirus, PD: post-dose.

**Table 4:** GMT ratios (by PRNT and RVP) in the FV-naïve cohort (per-protocol set).

		PRNT		RVP	
Time Point	Comparison	GMT Ratio (95% CI)	*p*-Value	GMT Ratio (95% CI)	*p*-Value
Pre-dose 1	5 μg vs. 2 μg 10 μg vs. 2 μg 10 μg vs. 5 μg	- - -	- - -	1.0 (0.7–1.3) 1.3 (1.0–1.8) 1.4 (1.1–1.8)	0.752 0.050 0.004
PD1	5 μg vs. 2 μg 10 μg vs. 2 μg 10 μg vs. 5 μg	1.59 (0.65–3.87) 2.05 (0.89–4.76) 1.29 (0.61–2.74)	0.301 0.092 0.500	1.7 (1.0–2.9) 3.4 (2.0–5.9) 2.0 (1.2–3.5)	0.054 <0.001 0.012
PD2	5 μg vs. 2 μg 10 μg vs. 2 μg 10 μg vs. 5 μg	1.76 (1.07–2.91) 3.27 (1.98–5.39) 1.85 (1.15–2.98)	0.027 0.000 0.012	1.9 (1.1–3.5) 4.0 (2.3–6.9) 2.1 (1.2–3.5)	0.031 <0.001 0.008

Abbreviations: CI: confidence interval; FV: flavivirus, GMT: geometric mean titer, PD: post-dose, PRNT: plaque reduction neutralization test, RVP: reporter virus particle assay.

## Data Availability

The datasets from the clinical study NCT03343626, will be made available within 3 months from initial request to researchers who provide a methodologically sound proposal. The datasets used and/or analyzed during the current study are available from the corresponding author on reasonable request. The data will be provided after its de-identification, in compliance with applicable privacy laws, data protection, and requirements for consent and anonymization.
